# Time-Matching Random Finite Set-Based Filter for Radar Multi-Target Tracking

**DOI:** 10.3390/s18124416

**Published:** 2018-12-13

**Authors:** Defu Jiang, Ming Liu, Yiyue Gao, Yang Gao, Wei Fu, Yan Han

**Affiliations:** 1 Laboratory of Array and Information Processing, Hohai University, Nanjing 210098, China; jiangdefu@hhu.edu.cn (D.J.); 23shi@hhu.edu.cn (Y.G.); fuwei@hhu.edu.cn (W.F.); hyan0525@hhu.edu.cn (Y.H.); 2 College of Energy and Electrical Engineering, Hohai University, Nanjing 210098, China; 20080039@hhu.edu.cn

**Keywords:** random finite sets, Bayesian filtering, sampling time diversity, radar multi-target tracking, generalized labeled multi-Bernoulli, probability hypothesis density

## Abstract

The random finite set (RFS) approach provides an elegant Bayesian formulation of the multi-target tracking (MTT) problem without the requirement of explicit data association. In order to improve the performance of the RFS-based filter in radar MTT applications, this paper proposes a time-matching Bayesian filtering framework to deal with the problem caused by the diversity of target sampling times. Based on this framework, we develop a time-matching joint generalized labeled multi-Bernoulli filter and a time-matching probability hypothesis density filter. Simulations are performed by their Gaussian mixture implementations. The results show that the proposed approach can improve the accuracy of target state estimation, as well as the robustness.

## 1. Introduction

Radar, with the capability of all-weather monitoring day and night, has been widely used in civil and military applications [1,2,3,4,5,6]. As an important part of the radar system, multi-target tracking (MTT) has received much research attention in the past few decades [7,8,9,10,11,12,13,14,15]. It aims at providing simultaneous estimation of the number of objects and their individual states based on a sequence of noisy measurements.

One of the main challenges in MTT is that the available measurements can come from real targets, as well as false targets such as clutters. This problem is handled by data association techniques in conventional MTT approaches such as multiple hypothesis tracking (MHT) [7,8,16], joint probabilistic data association (JPDA) [7,8,17], and probabilistic multiple hypothesis tracking (PMHT) [11,18,19,20]. However, data association is time consuming, especially when clutter density is high and the number of measurements is large. In addition, as noted elsewhere [19], it is unclear whether data association-based methods are mathematically consistent with the Bayesian paradigm.

Alternatively, the MTT problem has been recast in the Bayesian filtering paradigm using random finite set (RFS) models in recent years [18,21,22,23,24,25], which recursively propagates the filtering density of the multi-target state forward in time. The resulting optimal multi-target Bayesian filter has laid the foundation for developing many innovative multi-target filters such as the probability hypothesis density (PHD) filter [15,18], the cardinalized PHD (CPHD) filter [26], the multi-target multi-Bernoulli (MeMBer) filter [11], and the cardinality-balanced MeMBer (CBMeMBer) filter [27]. Without the requirement to generate explicit associations of measurements to targets, these approaches have been very popular in the MTT field. Since these filters rest on the premise that targets are indistinguishable, tracks must be formed via additional post-processing in applications requiring target trajectories. For this reason, labeled multi-Bernoulli (LMB), as well as generalized labeled multi-Bernoulli (GLMB) filters, which are based on the labeled random finite sets, were proposed in [28,29,30,31,32] to prove target trajectories.

In order to improve the performance of the RFS-based approaches in radar applications, we propose a time-matching filtering framework in this paper to deal with the problem induced by the sampling time diversity. In radar applications, the targets located at different places are usually detected at different times during a scan, so it is important to match the filtering time and the sampling times of measurements. Traditionally, time-matching is an essential component of radar MTTs, as exemplified by the time-stamp alignment process in conventional data association-based MTT approaches [7,8] and the time synchronization problem in multi-sensor multi-target information fusion [11]. However, until now, to the best of the authors’ knowledge, too little attention has been paid to this problem in the area of RFS-based single-sensor MTT. This is mainly because time matching has a relationship with data association problems, and the requirement of data association has been sidestepped in RFS-based approaches. Furthermore, in many cases, the diversity of sampling times is not obvious, which is also one factor that causes the difference to be ignored. In some cases, however, the situation can be very different. For example, when the surveillance area is very large and the target is far from the radar, it is necessary for the antenna to scan slowly, so that its beam can illuminate in each direction for a long time to obtain a relatively good signal-to-noise ratio (SNR). This leads to a wide difference in sampling time between different targets, so much so that we have to treat them differently.

In radar applications, the antenna can only detect targets in a limited area of the measurement space that the beam can cover. Therefore, we divide the measurement space into several small areas according to the sampling times and assume that the different regions are independent of each other. Based on this assumption, we model both the multi-target state and the measurements at each scan as special RFSs, whose elements are also RFSs, each corresponding to a specific sampling time. The time matching Bayesian filter framework including the state transition function and the likelihood function is developed directly from these models. Based on the new framework, we propose a time-matching joint-GLMB filter and a time-matching PHD filter. In the simulations, we use Gaussian mixtures (GMs) to approximate the GLMB distribution and the PHD to verify the performance of the two filters. The results show that the proposed methods can suppress the measurement errors caused by the diversity of sampling time, and thus improve the accuracy and robustness of multi-target state estimation.

The rest of this paper is organized as follows. Section 2 contains a brief background on RFS-based filtering, along with the PHD filter and joint-GLMB filter. Section 3 presents the time-matching RFS-based Bayesian filtering framework, as well as its PHD and joint-GLMB implementations. The simulation results are given in Section 4, followed by the conclusions of this study in Section 5.

## 2. Background

### 2.1. Random Finite Set and Bayesian Multi-Target Filter

In an MTT system, the state of any target is assumed to follow a Markov process on the state space $X\subset R^{n_{x}}$, where $R^{n}$ is the set of real vectors of length *n* and $n_{x}$ is the dimension of target states. Given a state $x_{k-1}$ at the (k−1)th time step, the Markov state transition density to the state $x_{k}$ at the *k*th time step is $f_{k|k-1}\left(x_{k}|x_{k-1}\right)$. The target states are partially observed in the measurement space $Z\subset R^{n_{z}}$, where $n_{z}$ is the measurement dimension, and the observation of each state is modeled by a likelihood function. Given a state $x_{k}$ at the *k*th time step, the likelihood that the measurement $z_{k}\in Z$ is generated by $x_{k}$ is $g_{k}\left(z_{k}|x_{k}\right)$.

In RFS-based filters, the multi-target states and multi-target measurements at the *k*th time step are modeled as finite sets:

(1) $$\begin{array}{c} X_{k}=\left\{x_{k}^{1},\ldots ,x_{k}^{N_{x,k}}\right\}\in F\left(X\right), \end{array}$$

(2) $$\begin{array}{c} Z_{k}=\left\{z_{k}^{1},\ldots ,z_{k}^{N_{z,k}}\right\}\in F\left(Z\right), \end{array}$$

where $N_{x,k}$ and $N_{z,k}$ are the target number and measurement number, respectively, while F(X) and F(Z) are the collections of all finite subsets of targets and measurements, respectively. Owning to the existing target birth and death, $X_{k}$ can be expressed as:

(3) $$\begin{array}{c} X_{k}\;=\;\left\{{⋃}_{x\in X_{k-1}}\;S_{k|k-1}\left(x\right)\right\}\cup \left\{{⋃}_{x\in X_{k-1}}\;B_{k|k-1}\left(x\right)\right\}\;\cup \;B_{k}, \end{array}$$

where $S_{k|k-1}\left(x\right)$ and $B_{k|k-1}\left(x\right)$ are the random finite sets (RFSs) of the survived targets and spawn targets from previous state $X_{k-1}$, respectively, and $B_{k}$ is the RFS of newly-born targets. Likely, the measurement set $Z_{k}$ is composed of:

(4) $$\begin{array}{c} Z_{k}=\left\{{⋃}_{x\in X_{k}}\;G_{k}\left(x\right)\right\}\cup \Gamma_{k}, \end{array}$$

where $G_{k}\left(x\right)$ is the RFS of measurements from detected targets and $\Gamma_{k}$ denotes the RFS of clutters.

The RFS theory enables the MTT to be expressed in the Bayesian multi-target filter. The prediction from time k−1 to time *k* is calculated by the Chapman–Kolmogorov equation:

(5) $$\begin{array}{c} \pi_{k|k-1}\left(X_{k}|Z^{\left(k-1\right)}\right)\;=\;\int \;f_{k|k-1}\left(X_{k}|X\right)\pi_{k-1}\left(X|Z^{\left(k-1\right)}\right)\delta X, \end{array}$$

where the integral is a set integrals defined for any function f:F(X)→R by [33]:

(6) $$\begin{array}{c} \int f\left(X\right)\delta X=\sum_{i=1}^{\infty }\frac{1}{i!}\int f\left(\left\{x_{1},\ldots ,x_{i}\right\}\right)d\left(x_{1},\ldots ,x_{i}\right). \end{array}$$

Here, $\pi_{k-1}\left(X|Z^{\left(k-1\right)}\right)$ denotes the multi-object density, $Z^{\left(k-1\right)}:Z_{1},\ldots ,Z_{k-1}$ is a history of previous measurement sets, and $f_{k|k-1}\left(X_{k}|X\right)$ denotes the multi-object transition kernel.

The update equation is given by the Bayes rule:

(7) $$\begin{array}{c} \pi_{k}\left(X_{k}|Z^{\left(k\right)}\right)\;=\;\frac{g_{k}\left(Z_{k}|X_{k}\right)\pi_{k|k-1}\left(X_{k}|Z^{\left(k-1\right)}\right)}{\int \;g_{k}\left(Z_{k}|X_{k}\right)\pi_{k|k-1}\;\left(X_{k}|Z^{\left(k-1\right)}\right)\mu_{s}\;\left(dX\right)}, \end{array}$$

where $g_{k}\left(Z_{k}|X_{k}\right)$ denotes the multi-object likelihood function.

### 2.2. Sampling Time Diversity in Radar Applications

In radar applications, the bandwidth of the antenna is limited, so targets in different directions (or sectors) are unlikely to be detected at the same time, leading to a diversity of sampling times. This diversity depends not only on where the target is, but also on other factors such as the scanning speed of the antenna and its scanning strategy. When the antenna scans slowly, the sampling times of targets may vary greatly. At the same time, the special strategy of an electronically-scanning antenna may lead to a large difference in the sampling times of close targets and make the times of those far away from each other similar.

In existing RFS-based filters, the targets are assumed to be generated at the same time, generally at the last moment of each scan. However, the targets may have left their measured positions at that time, and thus, the direct use of these measurements in the update process could result in additional measurement errors. As such, we aim at presenting a time-matching RFS-based filtering framework to handle this problem in the rest of this paper.

## 3. Time-Matching RFS-Based MTT

In this section, we first propose the time-matching RFS-based Bayesian filtering framework and then present a time-matching joint-GLMB filter and a time-matching PHD filter based on this paradigm.

### 3.1. Time-Matching Bayesian Filtering

In radar applications, there is a one-to-one correspondence between directions and sampling times. Thus, we can divide the surveillance area into numbers of directions according to the sampling times and give the assumptions as:

**Assumption** **1.** *Each target evolves and generates measurements independently of each other.*

**Assumption** **2.** *Each direction is scanned at most one time per sampling period.*

**Assumption** **3.** *Any target, in a sampling period, can only appear in one direction.*

Assumption 1 is very very common in MTT applications. Assumptions 2 and 3 may not hold in practice, but we can still use them to derive the following filtering framework. We present the detailed analyses of this problem in Remarks 2 and 3.

Based on Assumptions 2 and 3, we rewrite the multi-target state model in Equation (1) as:

(8) $$\begin{array}{c} X_{k}=\left\{\left(X_{k}^{1},t_{k}^{1}\right),\ldots ,\left(X_{k}^{N_{t,k}},t_{k}^{N_{t,k}}\right),\left(X_{k}^{N_{t,k}+1},t_{k}\right)\right\}\in F\left(F\left(X\right)\times T\right), \end{array}$$

where $\left\{t_{K}^{1},\ldots ,t_{k}^{N_{t,k}}\right\}$ is the set of distinct sampling times at scan *k*, $X_{k}^{i}$ denotes the state of the targets included in the direction corresponding to the *i*th sampling time $t_{k}^{i}$, $X_{k}^{N_{t,k}+1}$ denotes the state of undetected targets, $t_{k}$ denotes the end of scan k, and T denotes the sampling time space.

By combining Assumptions 1 and 3, we can assume that the elements of $X_{k}$ are independent of each other. Then, the posterior density of $X_{k}$ can be further expressed by the density product of its elements, as follows:

(9) $$\begin{array}{c} \pi_{k|k}\left(X_{k}|Z^{\left(k\right)}\right)=\prod_{i=1}^{N_{t,k}+1}\pi_{k|k}\left(X_{k}^{i}|Z^{\left(k\right)}\right), \end{array}$$

where $\left(X_{k}^{i}|Z^{\left(k\right)}\right)$ denotes the density of the target state contained in the direction corresponding to $t_{k}^{i}$.

Given the posterior at scan k−1, $\pi_{k-1|k-1}\left(X_{k-1}^{i}|Z^{\left(k-1\right)}\right)$, in the form of Equation (9), the prediction at scan *k* is given by:

(10) $$\begin{array}{l} \pi_{k|k-1}\left(X_{k}|Z^{\left(k-1\right)}\right)=\prod_{i=1}^{N_{t,k}+1}\pi_{k|k-1}\left(X_{k}^{i}|Z^{\left(k-1\right)}\right)\\ \;=\prod_{i=1}^{N_{t_{k}}+1}\int f_{k|k-1}\left(X_{k}^{i}|X\right)\pi_{k-1|k-1}\left(X|Z^{\left(k-1\right)}\right)\delta X, \end{array}$$

where $f_{k|k-1}\left(X_{k}^{i}|X_{k-1}\right)$ is the multi-target state transition kernel given by:

(11) $$\begin{array}{l} f_{k|k-1}\left(X_{k}^{i}|X_{k-1}\right)=f_{k|k-1}\left(\overset{N_{t_{k-1}}+1}{\underset{j=1}{⊎}}X_{k}^{i,j}|\overset{N_{t_{k-1}}+1}{\underset{j=1}{⊎}}\left\{\left(X_{k-1}^{j},t_{k-1}^{j}\right)\right\}\right)\\ \;=\prod_{j=1}^{N_{t_{k-1}}+1}f_{t_{k}^{i}|t_{k-1}^{j}}\left(X_{k}^{i,j}|X_{k-1}^{j}\right), \end{array}$$

where $f_{t_{k}^{i}|t_{k-1}^{j}}\left(X_{k}^{i,j}|X_{k-1}^{j}\right)$ is the standard state transition kernel from $t_{k-1}^{j}$ to $t_{k}^{i}$, and $X_{k}^{i,j}$ is the subset of $X_{k}^{i}$ evolved from $X_{k-1}^{j}$. Equation (11) results from a direct application of Assumptions 1 and 3.

Likely, the measurement model in Equation (2) can be rewritten as:

(12) $$\begin{array}{c} Z_{k}=\left\{\left(Z_{k}^{1},t_{k}^{1}\right),\ldots ,\left(Z_{k}^{N_{t,k}},t_{k}^{N_{t,k}}\right)\right\}\in F\left(F\left(Z\right)\times T\right), \end{array}$$

where $Z_{k}^{i}$ is the measurement set generated in the direction corresponding to $t_{k}^{i}$. Based on Assumption 1, the multi-target likelihood function is given by:

(13) $$\begin{array}{c} g_{k}\left(Z_{k}|X_{k}\right)=\prod_{i=1}^{N_{t,k}+1}g_{k}\left(Z_{k}^{i}|X_{k}^{i}\right), \end{array}$$

where $Z_{k}^{N_{t,k}+1}=\emptyset$. Then, given the prediction in Equation (10), the updated density is calculated by:

(14) $$\begin{array}{l} \pi_{k|k}\left(X_{k}|Z^{\left(k\right)}\right)=\frac{\prod_{i=1}^{N_{t,k}+1}g_{k}\left(Z_{k}^{i}|X_{k}^{i}\right)\pi_{k|k-1}\left(X_{k}^{i}|Z^{\left(k-1\right)}\right)}{\int \prod_{i=1}^{N_{t,k}+1}g_{k}\left(Z_{k}^{i}|X^{i}\right)\pi_{k|k-1}\left(X^{i}|Z^{\left(k-1\right)}\right)\delta X}\\ \;=\prod_{i=1}^{N_{t,k}+1}\frac{g_{k}\left(Z_{k}^{i}|X_{k}^{i}\right)\pi_{k|k-1}\left(X_{k}^{i}|Z^{\left(k-1\right)}\right)}{\int g_{k}\left(Z_{k}^{i}|X^{i}\right)\pi_{k|k-1}\left(X^{i}|Z^{\left(k-1\right)}\right)\delta X^{i}}\\ \;=\prod_{i=1}^{N_{t,k}+1}\pi_{k|k}\left(X_{k}^{i}|Z_{k}^{i}\right), \end{array}$$

where $\pi_{k|k}\left(X_{k}^{i}|Z_{k}^{i}\right)=\pi_{k|k}\left(X_{k}^{i}|Z_{k}^{i},Z^{\left(k-1\right)}\right)$ is the posterior related to time $t_{k}^{i}$. The final posterior probability density is the product of the densities from all distinct sampling times, the same as in Equation (9).

**Remark** **1.** *Again, based on Assumption 1, $\pi_{k|k}\left(X_{k}^{i}|Z_{k}^{i}\right)$ can be further expressed as:*

(15) $$\begin{array}{cc} \pi_{k|k}\left(X_{k}^{i}|Z_{k}^{i}\right) & =\pi_{k|k}\left(\overset{\left|Z_{k}^{i}\right|}{\underset{j=1}{⊎}}X_{k}^{i,j}|\overset{\left|Z_{k}^{i}\right|}{\underset{j=1}{⊎}}\left\{z_{k}^{i,j}\right\}\right)=\prod_{j=1}^{\left|Z_{k}^{i}\right|}\pi_{k|k}\left(X_{k}^{i,j}|\left\{z_{k}^{i,j}\right\}\right), \end{array}$$

*where $\left\{z_{k}^{i,j}\right\}$ is the jth measurement of $Z_{k}^{i}$. Then, the posterior can be given by:*

(16) $$\begin{array}{l} \pi_{k|k}\left(X_{k}|Z^{\left(k\right)}\right)=\pi_{k|k}\left(X_{k}^{ND}|\emptyset \right)\times \prod_{i=1}^{N_{t,k}+1}\prod_{j=1}^{\left|Z_{k}^{i}\right|}\pi_{k|k}\left(X_{k}^{i,j}|\left\{z_{k}^{i,j}\right\}\right)\\ \;=\pi_{k|k}\left(X_{k}^{ND}|\emptyset \right)\times \prod_{i=1}^{N_{z,k}}\pi_{k|k}\left(X_{k}^{i}|\left\{z_{k}^{i}\right\}\right)\\ \;=\prod_{i=1}^{N_{z,k}+1}\frac{g_{k}\left(\left\{z_{k}^{i}\right\}|X_{k}^{i}\right)\pi_{k|k-1}\left(X_{k}^{i}|Z^{\left(k-1\right)}\right)}{\int g_{k}\left(\left\{z_{k}^{i}\right\}|X^{i}\right)\pi_{k|k-1}\left(X^{i}|Z^{\left(k-1\right)}\right)\delta X^{i}}, \end{array}$$

*where $X_{k}^{ND}$ is the multi-target state of undetected targets, $X_{k}=X_{k}^{ND}⊎\left({⊎}_{i=1}^{N_{z,k}}X_{k}^{i}\right)$, $N_{z,k}$ is the number of all measurements collected at scan k, and $z_{k}^{i}$ is the ith measurement with the sampling time of $t_{k}^{i}$. It is more convenient to use Equation (16) than Equation (14) in some practical applications because, according to Equation (16), one can begin the calculation of the posterior as soon as any measurement is generated.*

**Remark** **2.** *In practice, it is possible for a direction to be scanned more than once during a scan period. This is common in the applications using an electronic scanning antenna. If a direction (on sector) of the surveillance area is likely to have targets or that direction has a relatively high priority, it may be scanned many times during a sampling period. Even so, the proposed filtering framework is still reasonable. If a direction is scanned many times in a sampling period, according to Assumption 3, we can assume that the targets contained in that direction remain the same for each scan. We can also assume that the scans are independent of each other because they occur at different times. Therefore, this problem can be thought as a multi-sensor multi-target problem [20]. The posterior of that direction can be calculated by recursively using the multi-target Bayesian filter:*

(17) $$\begin{array}{c} \pi_{k|k}\left(X_{k}^{i}|Z_{k}^{i,1},\ldots ,Z_{k}^{i,N_{S}^{i}}\right)=\frac{\pi_{t^{i,N_{S}^{i}}|t^{i,N_{S}^{i}}}\left(Z_{k}^{i,N_{S}^{i}}|X_{k}^{i},Z_{k}^{i,1},\ldots ,Z_{k}^{i,N_{S}^{i}-1}\right)\pi_{t^{i,N_{S}^{i}}|t^{i,N_{S}^{i}-1}}\left(X_{k}^{i}|Z_{k}^{i,1},\ldots ,Z_{k}^{i,N_{S}^{i}-1}\right)}{\pi_{t^{i,N_{S}^{i}}|t^{i,N_{S}^{i}}}\left(Z_{k}^{i,1},\ldots ,Z_{k}^{i,N_{S}^{i}}\right)}, \end{array}$$

*where $N_{S}^{i}$ is the scan number of the ith direction, $Z_{k}^{i,j}$ is the measurement set generated at the jth scan, $t^{i,N_{S}^{i}}$ and $t^{i,N_{S}^{i}-1}$ denote respectively the sampling times of $Z_{k}^{i,N_{S}^{i}}$ and $Z_{k}^{i,N_{S}^{i}-1}$, and the denominator is given by:*

(18) $$\begin{array}{c} \pi_{t^{i,N_{S}^{i}}|t^{i,N_{S}^{i}}}\left(Z_{k}^{i,1},\ldots ,Z_{k}^{i,N_{S}^{i}}\right)=\int \left(\begin{array}{c} \pi_{t^{i,N_{S}^{i}}|t^{i,N_{S}^{i}}}\left(Z_{k}^{i,N_{S}^{i}}|X^{i},Z_{k}^{i,1},\ldots ,Z_{k}^{i,N_{S}^{i}-1}\right)\\ \times \pi_{t^{i,N_{S}^{i}}|t^{i,N_{S}^{i}-1}}\left(X^{i}|Z_{k}^{i,1},\ldots ,Z_{k}^{i,N_{S}^{i}-1}\right) \end{array}\right)\delta X^{i}. \end{array}$$

*The density processing time for each direction is the time that the direction is last scanned. Then, the posterior density of this sampling period can still be expressed in the form of Equation (14) as:*

(19) $$\begin{array}{c} \pi_{k|k}\left(X_{k}|Z^{\left(k\right)}\right)=\pi_{k|k}\left(X_{k}^{ND}|\emptyset \right)\times \prod_{i=1}^{N_{D}}\pi_{k|k}\left(X_{k}^{i}|Z_{k}^{i,1},\ldots ,Z_{k}^{i,N_{S}^{i}}\right), \end{array}$$

*where $N_{D}$ denotes the number of directions from which measurements are generated. It can be found that the time-matching Bayesian filtering framework is much more flexible than the standard one. This makes it possible to deal with complex problems in radar applications. However, in fact, it is a part of our future work to use the time-matching framework to deal with the kind of problems described in this remark, so we do not consider these issues in the following section.*

**Remark** **3.** *The other thing that we need to note is that in practical applications, it is also possible for Assumption 3 to be not valid. When an electronic scanning antenna is used, targets from different directions may interact with each other during a sampling period. This is mainly because the scanning mode of the electronic scanning antenna is flexible, and it can scan with beam agility. Based on this fact, targets that have not been detected may run into directions that have been scanned before they are detected, leading to misdetection. The only way to deal with this problem is to set appropriate detection probabilities. Likewise, targets that have been detected may move to directions that have not been scanned, leading to the double-counting problem, which is common in multi-sensor function. The double-counting problem can result in two parallel tracks and waste computing resources. For this problem, we can detect and delete redundant tracks by using a track function process.*

In the rest of this section, we focus on proposing two RFS-based filters developed based on the new framework, i.e., the time-matching joint-GLMB filter and time-matching PHD filter.

### 3.2. Time-Matching Joint-GLMB Filter

In order to implement the Bayesian multi-target filter, the joint-GLMB filter models the target state as a δ-GLMB RFS [32], which is a labeled RFS with state space X and discrete label space L, distributed according to:

(20) $$\begin{array}{l} \pi (X)=\Delta \left(X\right)\sum_{\xi \in \Xi }w^{\left(\xi \right)}\left(L\left(X\right)\right){\left[p^{\left(\xi \right)}\right]}^{X}\\ \;=\Delta \left(X\right)\sum_{\xi \in \Xi ,I\in F(L)}w^{\left(I,\xi \right)}\delta_{I}\left[L\left(X\right)\right]{\left[p^{\left(\xi \right)}\right]}^{X}, \end{array}$$

where Ξ is a given discrete set representing a history of association maps, $w^{\left(I,\xi \right)}=w^{\left(\xi \right)}\left(I\right)$ is the weight of hypothesis L(X) with $\sum_{L\subseteq L}\sum_{\xi \in \Xi }w^{\left(\xi \right)}\left(L\right)=1$, $p^{\left(\xi \right)}\left(x,\ell \right)$ is the single-target distribution with $\int_{x\in X}p^{\left(\xi \right)}\left(x,\ell \right)dx=1$, the exponential of a real valued function *h* raised to a set *X* is defined as ${\left[h\right]}^{X}=\prod_{x\in X}h\left(x\right)$, $\delta_{Y}\left(X\right)$ is a generalization of the Kronecker delta that takes arbitrary arguments such as sets, vectors, integers, etc., denoted by:

(21) $$\begin{array}{c} \delta_{Y}\left[X\right]=\left\{\begin{array}{ll} \;1, & \mathrm{if}\;X=Y\\ \;0, & \mathrm{otherwise} \end{array}\right., \end{array}$$

and the distinct label indicator function is defined as $\Delta \left(X\right)≜\delta_{\left|X\right|}\left[|L\left(X\right)|\right]$, which ensures that the labels of X are distinct.

Given the prior δ-GLMB filtering density in Equation (20), the posterior δ-GLMB density in the joint-GLMB filter is given by:

(22) $$\begin{array}{c} \pi_{Z_{+}}\left(X\right)\propto \Delta \left(X\right)\sum_{I,\xi ,I_{+},\theta_{+}}w^{\left(I,\xi \right)}w_{Z_{+}}^{\left(I,\xi ,I_{+},\theta_{+}\right)}\delta_{I_{+}}\left[L\left(X\right)\right]{\left[p_{Z_{+}}^{\left(\xi ,\theta_{+}\right)}\right]}^{X}, \end{array}$$

where $Z_{+}$ is the measurement set collected at the current scan, I∈F(L), ξ∈Ξ, $I_{+}\in F\left(L_{+}\right)$, $\theta_{+}\in \Theta_{+}$, $\Theta_{+}$ is the set of positive 1-1maps $\theta_{+}:L\to \left\{0:|Z_{+}|\right\}$, $\theta_{+}$ denotes a track-measurement association hypothesis, and $w_{Z_{+}}^{\left(I,\xi ,I_{+},\theta_{+}\right)}$ is the weight used to update $w^{\left(I,\xi \right)}$.

According to the standard joint-GLMB filter [32], in order to update $w^{\left(I,\xi \right)}$, all possible track-measurement association hypotheses should be enumerated. This makes the joint-GLMB filter very compatible with time-matched filtering framework because track-to-measurement association is also the key to matching prediction and measurement times. According to Equation (22), $\theta_{+}$ is a track-measurement 1-1 map and specifies which tracks generated which measurements. Since each measurement is generated at a specific time, $\theta_{+}$ also specifies the sampling times of tracks, and so does ξ.

In order to take sampling time diversity into account, we augment the single-target state with the sampling time x=(x,ℓ,t). Let $t_{Z_{+}}^{\theta_{+}\left(\ell_{+}\right)}$ be the sampling time of track $\ell_{+}$ corresponding to $\theta_{+}$ at the current scan and $t^{\xi (\ell )}$ be the sampling time of track *ℓ* corresponding to ξ at the previous scan. The single-target state distribution in Equation (20) then is denoted as $p^{\left(\xi \right)}\left(x,\ell ,t^{\xi (\ell )}\right)$, and the updating weights in Equation (22) are given by:

(23) $$\begin{array}{c} w_{Z_{+}}^{\left(I,\xi ,I_{+},\theta_{+}\right)}=1_{\Theta_{+}\left(I_{+}\right)}\left(\theta_{+}\right){\left[1-{\bar{P}}_{S}^{\left(\xi \right)}\right]}^{I-I_{+}}{\left[{\bar{P}}_{S}^{\left(\xi \right)}\right]}^{I\cap I_{+}}\\ \;\times {\left[1-r_{B,+}^{\left(\theta_{+}\right)}\right]}^{B_{+}-L_{+}}{\left[r_{B,+}^{\left(\theta_{+}\right)}\right]}^{B_{+}\cap L_{+}}{\left[{\bar{\psi }}_{Z_{+}}^{\left(\xi ,\theta_{+}\right)}\right]}^{I_{+}}, \end{array}$$

where $1_{S}\left(\cdot \right)$ denotes the inclusive function of *S*, which is a generalization of the indicator function, and ${\bar{P}}_{S}^{\left(\xi \right)}\left(\ell \right)$ is the survival weight (or probability) of track *ℓ*, given by:

(24) $$\begin{array}{c} {\bar{P}}_{S}^{\left(\xi \right)}\left(\ell \right)={\bar{P}}_{S}^{\left(\xi \right)}\left(\ell ,t^{\xi (\ell )}\right)=\langle p^{\left(\xi \right)}\left(\cdot ,\ell ,t^{\xi (\ell )}\right),P_{S}\left(\cdot ,\ell ,t^{\xi (\ell )}\right)\rangle \end{array}$$

where $P_{S}\left(x,\ell ,t\right)$ is the survival probability of single-target state x, and for any functions g(x) and h(x), $\langle g(\cdot )h(\cdot )\rangle =\int g\left(x\right)h\left(x\right)dx$. The probability that a new target with label $\ell_{+}$ is born at time $t_{Z_{+}}^{\theta_{+}\left(\ell_{+}\right)}$ is denoted by $r_{B,+}^{\left(\theta_{+}\right)}\left(\ell_{+}\right)$, and $p_{B,+}\left(x_{+},\ell_{+},t_{Z_{+}}^{\theta_{+}\left(\ell_{+}\right)}\right)$ is its distribution. ${\bar{\psi }}_{Z_{+}}^{\left(\xi ,\theta_{+}\right)}\left(\ell_{+}\right)$ denotes the likelihood of track $\ell_{+}$, as well as the Bayesian normalization factor in Bayes rule, given by:

(25) $$\begin{array}{c} {\bar{\psi }}_{Z_{+}}^{\left(\xi ,\theta_{+}\right)}\left(\ell_{+}\right)={\bar{\psi }}_{Z_{+}}^{\left(\xi ,\theta_{+}\right)}\left(\ell_{+},t_{Z_{+}}^{\theta_{+}\left(\ell_{+}\right)}\right)=\langle {\bar{p}}_{+}^{\left(\xi \right)}\left(\cdot ,\ell_{+},t_{Z_{+}}^{\theta_{+}\left(\ell_{+}\right)}\right),\psi_{Z_{+}}^{\left(\theta_{+}\left(\ell_{+}\right)\right)}\left(\cdot ,\ell_{+},t_{Z_{+}}^{\theta_{+}\left(\ell_{+}\right)}\right)\rangle \end{array}$$

where ${\bar{p}}_{+}^{\left(\xi \right)}\left(x_{+},\ell_{+},t_{Z_{+}}^{\theta_{+}\left(\ell_{+}\right)}\right)$ is the predicted density of single-target state and $\psi_{Z_{+}}^{\left(\theta_{+}\left(\ell_{+}\right)\right)}\left(x_{+},\ell_{+},t_{Z_{+}}^{\theta_{+}\left(\ell_{+}\right)}\right)$ is the likelihood of single-target state related to the hypothesis of $\theta_{+}$. They are given respectively by:

(26) $$\begin{array}{ll} {\bar{p}}_{+}^{\left(\xi \right)}\left(x_{+},\ell_{+},t_{Z_{+}}^{\theta_{+}\left(\ell_{+}\right)}\right) & =1_{L(\ell_{+})}\frac{\langle f_{+}\left(x_{+},t_{Z_{+}}^{\theta_{+}\left(\ell_{+}\right)}|\cdot ,\ell_{+},t^{\xi (\ell_{+})}\right),P_{S}\left(\cdot ,\ell_{+},t^{\xi (\ell_{+})}\right)p^{\left(\xi \right)}\left(\cdot ,\ell_{+},t^{\xi (\ell_{+})}\right)\rangle }{{\bar{P}}_{S}^{\left(\xi \right)}\left(\ell_{+}\right)}\\ \; & +1_{B_{+}}\left(\ell_{+}\right)p_{B,+}\left(x_{+},\ell_{+},t_{Z_{+}}^{\theta_{+}\left(\ell_{+}\right)}\right), \end{array}$$

(27) $$\begin{array}{ll} \psi_{Z_{+}}^{\left(\theta_{+}\left(\ell_{+}\right)\right)}\left(x_{+},\ell_{+},t_{Z_{+}}^{\theta_{+}\left(\ell_{+}\right)}\right) & =\delta_{0}\left(\theta_{+}\left(\ell_{+}\right)\right)\left(1-P_{D}\left(x_{+},\ell_{+},t_{Z_{+}}^{\theta_{+}\left(\ell_{+}\right)}\right)\right)\\ \; & +\left(1-\delta_{0}\left(\theta_{+}\left(\ell_{+}\right)\right)\right)\frac{P_{D}\left(x_{+},\ell_{+},t_{Z_{+}}^{\theta_{+}\left(\ell_{+}\right)}\right)g\left(z_{+}^{\left(\theta_{+}\left(\ell_{+}\right)\right)}|x_{+},\ell_{+}\right)}{\kappa (z_{+}^{\left(\theta_{+}\left(\ell_{+}\right)\right)})}, \end{array}$$

where $f_{+}\left(x_{+},t_{Z_{+}}^{\theta_{+}\left(\ell_{+}\right)}|\cdot ,\ell_{+},t^{\xi (\ell_{+})}\right)$ denotes the kernel describing target transition from time $t^{\xi (\ell_{+})}$ to $t_{Z_{+}}^{\theta_{+}\left(\ell_{+}\right)}$, the detection probability is denoted by $P_{D}\left(x_{+},\ell_{+},t_{Z_{+}}^{\theta_{+}\left(\ell_{+}\right)}\right)$, $z_{+}^{\left(\theta_{+}\left(\ell_{+}\right)\right)}$ denotes the measurement generated at time $t_{Z_{+}}^{\theta_{+}\left(\ell_{+}\right)}$, $g(z_{+}^{\left(\theta_{+}\left(\ell_{+}\right)\right)}|x_{+},\ell_{+})$ denotes the likelihood that $z_{+}^{\left(\theta_{+}\left(\ell_{+}\right)\right)}$ is generated from state $\left(x_{+},\ell_{+},t_{Z_{+}}^{\theta_{+}\left(\ell_{+}\right)}\right)$, and $\kappa (z_{+}^{\left(\theta_{+}\left(\ell_{+}\right)\right)})$ is the probability that $z_{+}^{\left(\theta_{+}\left(\ell_{+}\right)\right)}$ is generated by clutters.

Given the normalization factor in Equation (25), predicted density in Equation (26), and likelihood in Equation (27), the updated density of the single-target state is calculated by Bayes rule as follows:

(28) $$\begin{array}{c} p_{Z_{+}}^{\left(\xi ,\theta_{+}\right)}\left(x_{+},\ell_{+}\right)=p_{Z_{+}}^{\left(\xi ,\theta_{+}\right)}\left(x_{+},\ell_{+},t_{Z_{+}}^{\theta_{+}\left(\ell_{+}\right)}\right)=\frac{{\bar{p}}_{+}^{\left(\xi \right)}\left(x_{+},\ell_{+},t_{Z_{+}}^{\theta_{+}\left(\ell_{+}\right)}\right)\psi_{Z_{+}}^{\left(\theta_{+}\left(\ell_{+}\right)\right)}\left(x_{+},\ell_{+},t_{Z_{+}}^{\theta_{+}\left(\ell_{+}\right)}\right)}{{\bar{\psi }}_{Z_{+}}^{\left(\xi ,\theta_{+}\right)}\left(\ell_{+}\right)}. \end{array}$$

**Remark** **4.** *It is worth noticing that the above algorithm is directly developed from the joint-GLMB filter proposed in [32]. If the sampling time diversity is ignored and assuming that targets are detected at the end of each scan, a constant transition interval will be used in the prediction in Equation (26), which is equal to the sampling period. As a result, the proposed joint-GLMB filter will collapse to the standard one in [32].*

Like other GLMB-based filters, the joint-GLMB filter can provide a closed-form solution for the multi-target Bayesian filter. However, it is somewhat computationally expensive, especially when the number of measurements is large. Compared with the joint-GLMB filter, the PHD filter has a better real-time performance, making it more suitable for scenes with high real-time requirements. As such, we propose a time-matching PHD filter in the next section.

### 3.3. Time-Matching PHD Filter

The PHD filter propagates the first moment of multi-target density, i.e., PHD, during each recursion [18]. The PHD is expressed as:

(29) $$\begin{array}{c} D\left(x\right)=\int \pi \left(x\cup W\right)\delta W=\int_{X∋x}\pi \left(X\right)\delta X. \end{array}$$

If we neglect the sampling time diversity, the posterior at scan *k* can be further expressed as:

(30) $$\begin{array}{l} D_{k|k}\left(x|Z^{\left(k\right)}\right)=\int_{X∋x}\prod_{i=1}^{N_{t,k}+1}\pi_{k|k}\left(X_{k}^{i}|Z_{k}^{i}\right)\delta X\\ \;=\sum_{j=1}^{N_{t,k}+1}\int_{X_{k}^{j}∋x}\prod_{i=1}^{N_{t,k}+1}\pi_{k|k}\left(X_{k}^{i}|Z_{k}^{i}\right)\delta X_{k}^{j}\\ \;=\sum_{i=1}^{N_{t,k}+1}\int_{X_{k}^{j}∋x}\pi_{k|k}\left(X_{k}^{i}|Z_{k}^{i}\right)\delta X_{k}^{i}\\ \;=D_{k|k}^{ND}\left(x\right)+\sum_{i=1}^{N_{t,k}}D_{k|k}\left(x|Z_{k}^{i}\right), \end{array}$$

where $D_{k|k}^{ND}\left(x\right)=\left(1-P_{D,k}\left(x\right)\right)D_{k|k-1}\left(x\right)$ is the PHD of undetected targets. Equation (30) results from an application of Equation (14) and the fact that targets in different directions are independent of each other. Considering the diversity of sampling time, we can further conclude that the posterior of scan *k* can be expressed as a PHD set given by:

(31) $$\begin{array}{c} D_{k|k}\left(x|Z^{\left(k\right)}\right)=\left\{D_{k|t_{k}^{1}}\left(x\right),\ldots ,D_{k|t_{k}^{N_{t,k}}}\left(x\right),D_{k|t_{k}}^{ND}\left(x\right)\right\}, \end{array}$$

where $D_{k|t_{k}^{i}}\left(x\right)$ is the updated PHD at time $t_{k}^{i}$, $t_{k}$ is the end of the scan, undetected PHD is $D_{k|t_{k}}^{ND}\left(x\right)=\left(1-P_{D,t_{k}}\left(x\right)\right)D_{t_{k}|k-1}\left(x\right)$, and $D_{t_{k}|k-1}\left(x\right)$ is the predicted PHD at time $t_{k}$.

Given the posterior at scan k−1 in the form of Equation (31), the predicted PHD at time $t_{k}^{i}$ is given by:

(32) $$\begin{array}{c} D_{t_{k}^{i}|k-1}\left(x\right)=b_{t_{k}^{i}|k-1}\left(x\right)+\sum_{t}\int F_{t_{k}^{i}|t}\left(x|x^{\prime }\right)D_{k-1|t}\left(x^{\prime }\right)dx^{\prime }, \end{array}$$

where $b_{t_{k}^{i}|k-1}\left(x\right)$ is the PHD of newly-born targets and $F_{t_{k}^{i}|t}\left(x|x^{\prime }\right)$ is the “pseudo-Markov density”, which is calculated by:

(33) $$\begin{array}{c} F_{t_{k}^{i}|t}\left(x|x^{\prime }\right)=b_{t_{k}^{i}|t}\left(x|x^{\prime }\right)+P_{S,t_{k}^{i}}\left(x^{\prime }\right)f_{t_{k}^{i}|t}\left(x|x^{\prime }\right), \end{array}$$

where $b_{t_{k}^{i}|t}\left(x|x^{\prime }\right)$ is the PHD of the targets spawned from the previous state $x^{\prime }$, $P_{S,t_{k}^{i}}\left(x^{\prime }\right)$ is the survival probability, and $f_{t_{k}^{i}|t}\left(x|x^{\prime }\right)$ is the single-target transition kernel.

Then, the updated PHD at time $t_{k}^{i}$ is given by:

(34) $$\begin{array}{c} D_{k|t_{k}^{i}}\left(x\right)=\sum_{z\in Z_{k}^{i}}\frac{P_{D,t_{k}^{i}}\left(x\right)g_{z,t_{k}^{i}}\left(x\right)D_{t_{k}^{i}|k-1}\left(x\right)}{\lambda_{t_{k}^{i}}c_{t_{k}^{i}}\left(z\right)+D_{t_{k}^{i}|k-1}\left[P_{D,t_{k}^{i}}g_{z,t_{k}^{i}}\right]}, \end{array}$$

where $P_{D,t_{k}^{i}}$ is the probability of detection, $g_{z,t_{k}^{i}}\left(x\right)$ is the single-target likelihood, $\lambda_{t_{k}^{i}}$ and $c_{t_{k}^{i}}\left(z\right)$ are respectively the clutter rate and clutter distribution, and h(x), D[h]=∫h(x)D(x). Finally, in order to extract multi-target state estimation, one needs first to estimate the number of existing targets, which is the sum of the integrals of the updated PHDs at all sampling times, and then find the ${\bar{N}}_{x,k}$ states that correspond to the largest local maxima of those PHDs.

Again, if targets are assumed to be detected at the same time (or at the end of each scan), the posterior PHD at scan *k* will be given by:

(35) $$\begin{array}{l} D_{k|k}\left(x|Z^{\left(k\right)}\right)=D_{k|t_{k}}^{ND}\left(x\right)+\sum_{i=1}^{N_{t,k}}D_{k|t_{k}^{i}}\left(x\right)\\ \;=D_{k|t_{k}}^{ND}\left(x\right)+\sum_{i=1}^{N_{t,k}}\sum_{z\in Z_{k}^{i}}\frac{P_{D,t_{k}^{i}}\left(x\right)g_{z,t_{k}^{i}}\left(x\right)D_{t_{k}^{i}|k-1}\left(x\right)}{\lambda_{t_{k}^{i}}c_{t_{k}^{i}}\left(z\right)+D_{t_{k}^{i}|k-1}\left[P_{D,t_{k}^{i}}g_{z,t_{k}^{i}}\right]}\\ \;=\left(1-P_{D,k}\left(x\right)\right)D_{k|k-1}\left(x\right)+\sum_{z\in Z_{k}}\frac{P_{D,k}\left(x\right)g_{z}\left(x\right)D_{k|k-1}\left(x\right)}{\lambda_{k}c_{k}\left(z\right)+D_{k|k-1}\left[P_{D,k}g_{z}\right]}, \end{array}$$

which is equal to the original PHD filter in [18].

**Remark** **5.** *In practice, it is more convenient to use Equation (16) than Equation (14) to calculate the posterior. However, such a multi-prediction model may cause the computational complexity of the filter to become very large as the number of measurements increases. For this problem, the gating techniques [22,34,35,36] can be used to eliminate the track-measurement association hypotheses, and the algorithms of post-processing can be used to prune the posterior to reduce the amount of calculation in the next prediction process.*

## 4. Simulations

In this section, simulations are designed to demonstrate the efficiencies of the proposed time-matching RFS-based filters. These filters are implemented by their GM implementations, and the results are compared with those of the standard joint-GLMB filter and the standard PHD filter. The simulations are performed in four different scenarios as follows.

### 4.1. Scenarios

**Figure 1:**
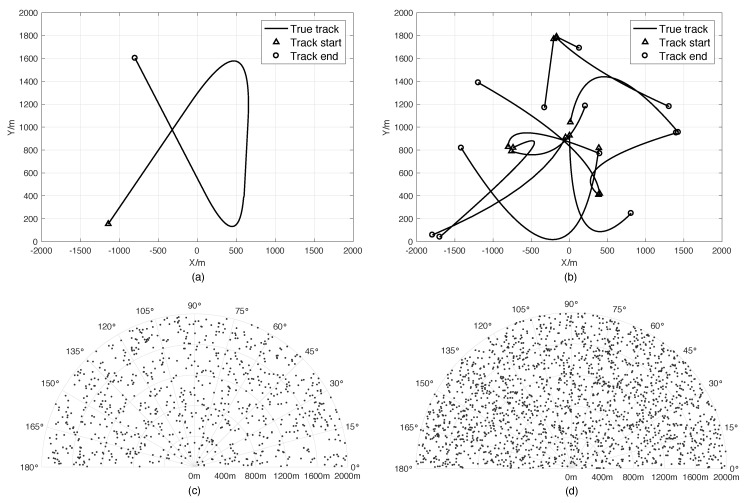
True trajectories of targets and clutters. (**a**) Target trajectories in Scenarios 1 and 2. (**b**) Target trajectories in Scenarios 3 and 4. (**c**) Clutter distribution in Scenarios 1 and 2. (**d**) Clutter distribution in Scenarios 3 and 4.

**Figure 2:**
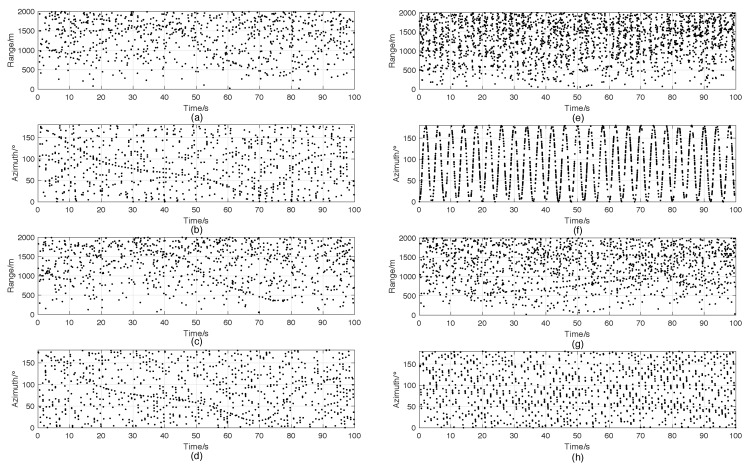
Measurements of one trail. (**a**) Range components of the measurements in Scenario 1. (**b**) Azimuth components of the measurements in Scenario 1. (**c**) Range components of the measurements in Scenario 2. (**d**) Azimuth components of the measurements in Scenario 2. (**e**) Range components of the measurements in Scenario 3. (**f**) Azimuth components of the measurements in Scenario 3. (**g**) Range components of the measurements in Scenario 4. (**h**) Azimuth components of the measurements in Scenario 4.

Scenario 1: In this scenario, a radar with a mechanical scanning radar scans a semicircle region with the range of 2000m during the interval of 100s. There is one target in the field of view (FOV). The true trajectory is drawn in Figure 1a. It starts moving at the position of (−1200 m, 100 m) at 0s and ending at (−801m,1602m) at 100s. The target swerves twice during the interval. One happens at 21s, and the other happens at 61s. The radar is located at (0m,0m), scanning bidirectionally at the speed of 180°/s, i.e., it scans repeatedly clockwise from 180°–0° and then anticlockwise from 0°–180°. The number of clutters is Poisson distributed with the clutter rate of 10, and each clutter is uniformly distributed in FOV, as shown in Figure 1c. The range resolution and azimuth resolution of the sensor are 10m and 1°, respectively, which are the same in other scenarios. Figure 2a,b shows the measurements versus time in the range and azimuth components separately.

**Figure 3:**
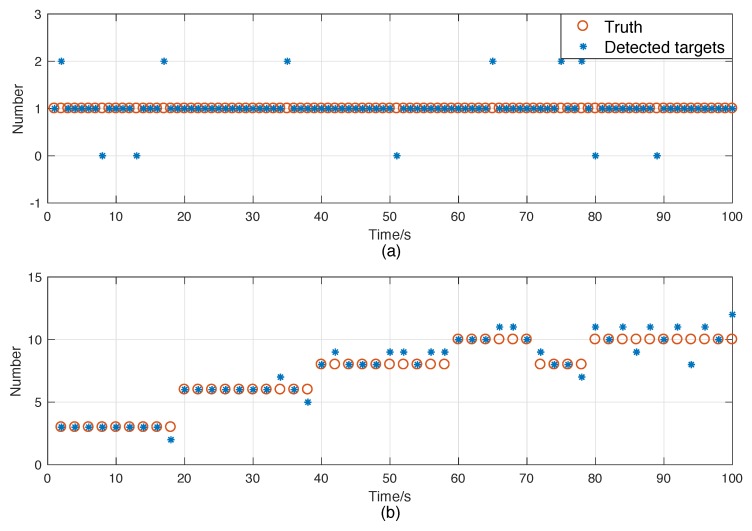
Number of detected targets. (**a**) Scenario 2. (**b**) Scenario 4.

Scenario 2: This scenario is similar to Scenario 1, except that the antenna is electronic scanning. In this case, we divide the surveillance area into twenty sectors and design the radar to scan in the order shown in Table 1. Figure 2c,d shows the measurements versus time in the range and azimuth components separately, and Figure 3a shows the number of detected targets at each scan.

Scenario 3: In this scenario, there are twelve targets moving in FOV. Figure 1b illustrates the true target trajectories, and the initial states and lifetimes of these targets are listed in Table 2. We use the same mechanical scanning radar as in Scenario 1, but the scanning speed is set to 90°/s, i.e., each scan costs 2s. The clutter is also uniformly distributed in FOV, whose expected number is set to 50 as shown in Figure 1d. Figure 2e,f shows the corresponding measurements in the range and azimuth components.

Scenario 4: In Scenario 4, the target trajectories and clutters are similar to those in Scenario 3, but we use an electronic scanning antenna with the scanning speed of 90°/s. The sensor scans according to the order shown in Table 1. The measurements in the range and azimuth components are shown in Figure 2g,h, respectively. Figure 3b shows the cardinality of the targets detected per scan.

The detection probability of the sensor in each scenario is set to 85%, as the false alarm threshold may cause the targets whose echo intensities are not that high to fail to generate measurements.

### 4.2. Target Tracking Setup

Without loss of generality, the single-target motion model presented in [37] is used for all filters, which is given by:

(36) $$\begin{array}{c} x_{k}=\left(F_{k|k-1}⊗I_{d}\right)x_{k-1}+w_{k}, \end{array}$$

where $x_{k}={\left[x_{k},y_{k},{\dot{x}}_{k},{\dot{y}}_{k},{\ddot{x}}_{k},{\ddot{y}}_{k}\right]}^{T}$, ${\left[x_{k},y_{k}\right]}^{T}$ is the position; ${\left[{\dot{x}}_{k},{\dot{y}}_{k}\right]}^{T}$ and ${\left[{\ddot{x}}_{k},{\ddot{y}}_{k}\right]}^{T}$ are respectively the velocity and acceleration, $I_{d}$ is the identity matrix of dimension *d*, the notation A⊗B denotes the Kronecker product of matrices *A* and *B*, and $w_{k}$ is the zero mean Gaussian process noise with covariance $\Delta_{k|k-1}=Q_{k|k-1}⊗I_{d}$. Here, d=2 and $F_{k|k-1}$ and $Q_{k|k-1}$ are given by:

(37) $$\begin{array}{c} F_{k|k-1}=\left[\begin{array}{ccc} 1 & T_{s} & \frac{1}{2}T_{s}^{2}\\ 0 & 1 & T_{s}\\ 0 & 0 & e^{\frac{-T_{s}}{\tau }} \end{array}\right] \end{array}$$

and:

(38) $$\begin{array}{c} Q=\Sigma^{2}\left(1-e^{\frac{-2T_{s}}{\vartheta }}\right)\left[\begin{array}{ccc} 0 & 0 & 0\\ 0 & 0 & 0\\ 0 & 0 & 10 \end{array}\right], \end{array}$$

respectively, where $T_{s}$ is the sampling period, Σ is the scalar acceleration standard deviation, and ϑ is the maneuver correlation time. In this paper, Σ and ϑ are set to 0.1 and 1s, respectively.

The corresponding single-target observation model is given by [37]:

(39) $$\begin{array}{c} z_{k}=\left[\begin{array}{c} r(\left(H_{k}⊗I_{d}\right)x_{k})\\ \theta (\left(H_{k}⊗I_{d}\right)x_{k}) \end{array}\right]+\left[\begin{array}{c} r(e_{k})\\ \theta (e_{k}) \end{array}\right], \end{array}$$

where $r(\left(H_{k}⊗I_{d}\right)x_{k})$ and $\theta (\left(H_{k}⊗I_{d}\right)x_{k})$ denote the range and bearing of state $x_{k}$, respectively, $H_{k}=\left[1\;0\;0\right]$ denotes the observation matrix, and $e_{k}$ is the white Gaussian noise with the covariance of diag([9,0.01]).

A total of 200 Monte Carlo (MC) trails were preformed for each scenario. The results are presented in terms of the multi-target measure optimal subpattern assignment metric (OSPA) [38], cardinality, and time costs. In the assessments, the cut-off and order parameters of the OSPA are set to c=200 and p=1, respectively.

### 4.3. Result Analysis of Scenario 1

**Figure 4:**
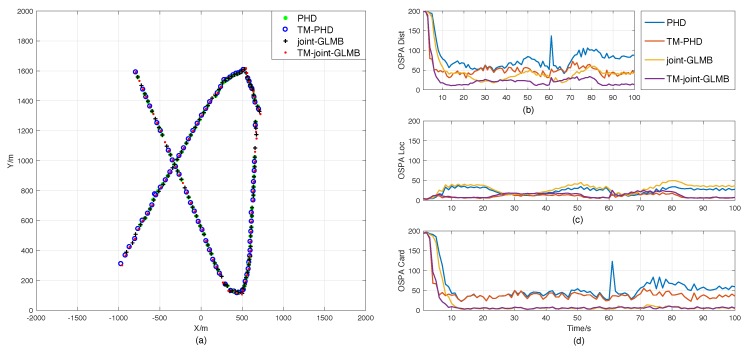
Result comparisons in Scenario 1. (**a**) Position estimations of the original random finite set (RFS)-based filters and the time-matching filters in one trial. (**b**) Optimal subpattern assignment metric (OSPA) errors. (**c**) Location components of OSPA. (**d**) Cardinality components of OSPA. PHD, probability hypothesis density; GLMB, generalized labeled multi-Bernoulli filter.

Scenario 1 was designed to demonstrate the efficiency of the time-matching Bayesian filtering framework. The result comparison of the PHD, time-matching PHD, joint-GLMB, and time-matching joint-GLMB filters is presented in Figure 4. It shows that the time-matching RFS-based filter is better than the standard ones in single-target tracking.

Figure 4a presents the position estimations of the four filters in one trail. It shows that the trajectory integrities of the proposed filters are similar to those of the standard RFS-based filters, which is consistent with the results shown in Figure 4d, that they have similar estimation accuracies of cardinality. Therefore, the OSPA differences between the sets of filters shown in Figure 4b are mainly caused by the differences of the location estimation errors presented in Figure 4c. This is in agreement with what was mentioned in Section 2.2, that ignoring the sampling time diversity may cause additional measurement errors in the standard RFS-based filters.

Actually, the errors of sampling time may also affect the accuracy of cardinality estimation. As shown in Figure 4d, the performance of the cardinality estimation of the standard PHD filter becomes worse after 61 s. The main reason is the increase in the velocity of target after 61 s, which can enhance the measurement errors caused by the time errors. As a result, the standard PHD filter fails to track the target in some trails.

On the contrary, owning to time matching, the proposed filters can handle the problem properly, and the estimation errors kept at relatively low and stable levels all the time.

### 4.4. Result Analysis of Scenario 2

**Figure 5:**
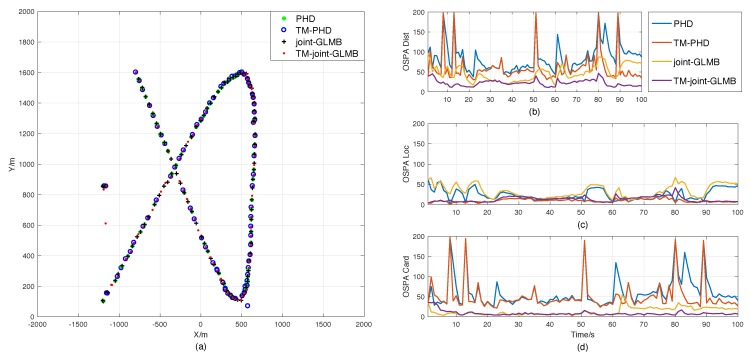
Result comparisons in Scenario 2. (**a**) Position estimations of the original RFS-based filters and the time-matching filters in one trial. (**b**) OSPA errors. (**c**) Location components of OSPA. (**d**) Cardinality components of OSPA.

Scenario 2 was designed to evaluate the performances of the proposed filters when the double-counting problem described in Remark 3 happens. Figure 5 presents the result comparisons of the four filters. Even though their error curves fluctuated a few times, they could still successfully track the target. Again, as the time-matching RFS-based filters consider the sampling time diversity, they still have better estimation performance than the standard RFS-based filters, as shown in Figure 5b, which is mainly caused by the differences in location estimation shown in Figure 5c. The differences in cardinality estimation shown in Figure 5d are similar to those in Scenario 1.

It is noteworthy that the error curves fluctuated the most at 8s, 13s, 51s, 80s, and 89s. As shown in Figure 3a, these times are exactly when misdetection happens. It seems that only misdetection affected the performance of the filter, but that is not the case. In fact, the double-counting also contributed to the deterioration of state estimation performance. The main cause of this phenomenon is that there was only one target in this scenario. This makes the normalized estimation error of underestimating one target much larger than that of overestimating one target. Thus, as the number of targets increase, the error curve of estimation, especially the cardinality estimation, will become smoother, which can be found in the multi-target tracking scenarios.

### 4.5. Result Analysis of Scenarios 3 and 4

**Figure 6:**
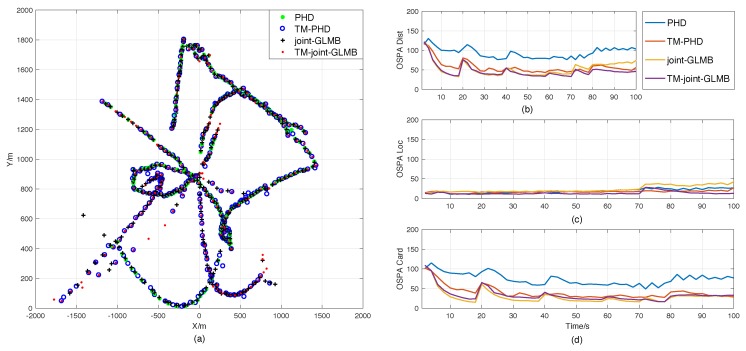
Result comparisons in Scenario 3. (**a**) Position estimations of the original RFS-based filters and the time-matching filters in one trial. (**b**) OSPA errors. (**c**) Location components of OSPA. (**d**) Cardinality components of OSPA.

**Figure 7:**
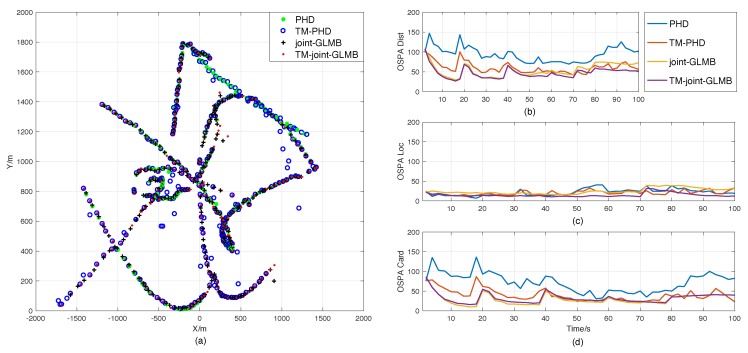
Result comparisons in Scenario 4. (**a**) Position estimations of the original RFS-based filters and the time-matching filters in one trial. (**b**) OSPA errors. (**c**) Location components of OSPA. (**d**) Cardinality components of OSPA.

Scenario 3 and 4 were designed to evaluate the multi-target tracking performances of the proposed filters in complex environments. Their estimation result comparisons are shown in Figure 6 and Figure 7, respectively. It shows that time-matching Bayesian filtering framework can also enhance the performance of the RFS-based filters in MTT. We should note that the improved PHD filter not only outperformed the standard PHD filter, but also exceeded the joint-GLMB filter in state estimation for some times. In addition, as expected, the error curves in Scenario 4 in Figure 6 are much smoother than those in Scenario 2, even though misdetection and double-counting happened more often, as shown in Figure 3.

As mentioned in Remark 5, owing to the multi-prediction model, the time-matching based method is more computationally expense. To handle this problem, one can use gating techniques. In this work, for the sake of simplicity, we applied the gating method based on the distances between measurements and the projections of single-target states on the space of measurement. The distance threshold was calculated by:

(40) $$\begin{array}{c} d_{lim}=∥2\times T_{s}\times v_{max}{∥}_{2}+w_{max} \end{array}$$

where $v_{max}$ is the maximum of target speed set as $v_{max}=100\;m/s$ and $w_{max}$ is the maximum error caused by observation inaccuracy set as $w_{max}=0\;m$.

**Figure 8:**
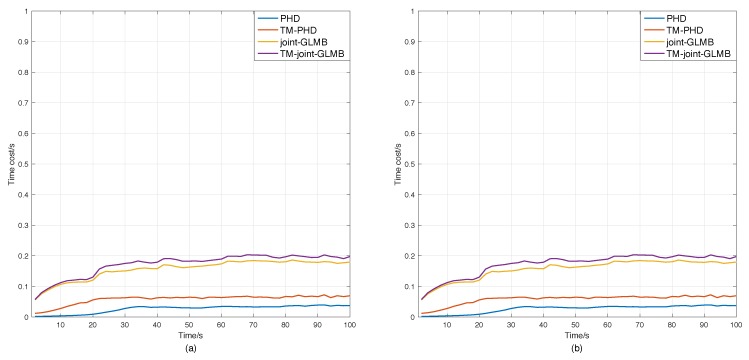
Time costs of the original RFS-based filters and the time-matching filters. (**a**) Scenario 3. (**b**) Scenario 4.

Figure 8 presents the time costs of the filters in Scenarios 3 and 4. It is shown that based on gating, the proposed time-matching RFS-based filters can achieve similar real-time performances as the standard ones.

In summary, all simulation results demonstrated that the performance of the time-matching RFS-based filters was better than the basic RFS-based filters, not only in cardinality estimation, but also in position estimation. By choosing a proper gating technique, one can also achieve similar real-time performance.

## 5. Conclusions

In this paper, we propose a time-matching RFS-based MTT framework in which the sampling time diversity of the radar MTT is considered. This makes it possible to use RFS-based filters to deal with the complex radar MTT problems. Based on this framework, we also propose a time-matching joint-GLMB filter and a time-matching PHD filter. By the GM approximations, we evaluate their performances in many real-world simulations. The results demonstrate that the proposed methods can handle the problems caused by sampling time diversity and provide enhanced filtering performances in state estimation. However, some challenges still remain in the proposed filter, including the multi-scan problems described in Remark 2 and the relatively high computational cost for the time-matching joint-GLMB filter. In further study, we will try to address these issues. Moreover, how to apply the proposed time-matching RFS-based MTT framework to extended target tracking is also our focus in future work. 

## Figures and Tables

**Table 1:** Scanning order of the electronic scanning antenna in Scenarios 2 and 4.

**Sector No.**	**1**	**2**	**3**	**4**	**5**	**6**	**7**	**8**	**9**	**10**
Left border	0°	9°	18°	27°	36°	45°	54°	63°	72°	81°
Right border	9°	18°	27°	36°	45°	54°	63°	72°	81°	90°
Scanning order	1	3	5	7	9	11	13	15	17	19
**Sector No.**	**11**	**12**	**13**	**14**	**15**	**16**	**17**	**18**	**19**	**20**
Left border	90°	99°	108°	117°	126°	135°	144°	153°	162°	171°
Right border	99°	108°	117°	126°	135°	144°	153°	162°	171°	180°
Scanning order	2	4	6	8	10	12	14	16	18	20

**Table 2:** List of initial target states.

Target Index	Lifetime (s)	Initial States (m, m/s, m/s${}^{2}$, m, m/s, m/s${}^{2}$)
# 1	(1,70)	[0,0,0.33,0,−37,0.75]
# 2	(1,100)	[400,−10,0.4,−600,5,0.01]
# 3	(1,70)	[−800,20,−0.16,−200,−5,0.3]
# 4	(20,100)	[400,−7,−0.37,−100,−42,1]
# 5	(20,100)	[400,−1.4,−0.44,−600,10,0.05]
# 6	(20,100)	[0,5.5,0.29,0,22,−0.55]
# 7	(40,100)	[−800,32,−1.5,−200,11,−0.75]
# 8	(40,100)	[−200,15,0.3,800,−10,0]
# 9	(60,100)	[−800,−3,1.5,−200,15,−0.75]
# 10	(60,100)	[−200,−3,0,800,−15,0]
# 11	(80,100)	[0,−20,−5.6,0,−45,0.2]
# 12	(80,100)	[−200,15,0,800,−5,0]
